# Marginal and Conditional Confounding Using Logits

**DOI:** 10.1177/0049124121995548

**Published:** 2021-04-09

**Authors:** Kristian Bernt Karlson, Frank Popham, Anders Holm

**Affiliations:** 1 Department of Sociology, 4321 University of Copenhagen, Denmark; 2 MRC/CSO Social and Public Health Sciences Unit, 3526 University of Glasgow, United Kingdom; 3 Department of Sociology, Social Science Centre, The University of Western Ontario, London, Ontario, Canada

**Keywords:** logit, odds ratio, confounding, mediation, standardization

## Abstract

This article presents two ways of quantifying confounding using logistic response models for binary outcomes. Drawing on the distinction between marginal and conditional odds ratios in statistics, we define two corresponding measures of confounding (marginal and conditional) that can be recovered from a simple standardization approach. We investigate when marginal and conditional confounding may differ, outline why the method by Karlson, Holm, and Breen recovers conditional confounding under a “no interaction”-assumption, and suggest that researchers may measure marginal confounding by using inverse probability weighting. We provide two empirical examples that illustrate our standardization approach.

A widespread practice among social scientists is to compare the regression coefficients of the same predictor variable between models successively adding covariates. Changes in the coefficients are taken to reflect confounding or mediation caused by adding the covariates. Yet, in nonlinear probability models such as the logit model, the practice of comparing coefficients is hampered by a rescaling bias resulting from the identifying assumptions of these models (Breen, Karlson, and Holm 2018; Mood 2010; Winship and Mare 1984). Karlson, Holm, and Breen (2012; KHB) proposed a method that provides coefficient comparisons free of this rescaling bias. Their method exploits properties of orthogonalized predictors to estimate a logit model that yields the unadjusted logit coefficient (unadjusted for covariates) measured on the scale of the logit model adjusting for covariates. This coefficient is subsequently compared with the adjusted coefficient, thereby removing the bias caused by rescaling.

The issue of rescaling bias is known under different headings outside the social sciences. In epidemiology, the bias is known as the noncollapsibility of odds ratios (Greenland, Robbins, and Pearl 1999). In statistics, researchers distinguish between marginal and conditional odds ratios (Zeger, Liang, and Albert 1988). The marginal odds ratio gauges the average population response to a unit change in the predictor, that is, it is an average effect evaluated over the specific composition of the population under consideration. The conditional odds ratio measures the effect for specific individuals or specific groups of individuals (i.e., it is subject-specific). It measures individuals’ or individual groups’ response to a unit change in the predictor. Marginal and conditional odds ratios thus measure different quantities and, for this reason, cannot be directly compared. Comparing them would be the equivalent of comparing coefficients measured on different scales.

In this article, we demonstrate that confounding using logits—as defined by comparing unadjusted and adjusted effects—can be expressed in both marginal and conditional forms. We present a simple nonparametric standardization approach that allows researchers to measure both forms of confounding using marginal predictions from a logit model. We explain how the interpretations of the two types of confounding differ, and we provide a simulated example to show a situation under which the two will differ. We also show that the methodology by Karlson et al. (2012) gauges conditional confounding under an assumption of no interaction effect between the predictor and the confounder in the logit model. To illustrate the differences between marginal and conditional confounding, we present two empirical examples. Whereas the first examines how the intergenerational occupational association changes once we adjust for offspring’s educational attainment, the second examines how income mediates the racial gap in the attitude toward whether whites are committed to the fair and equal treatment of all groups in society. We conclude the article with a discussion of why marginal confounding may be preferred over conditional confounding in applied social science research.

## Marginal and Conditional Odds Ratios

### The Marginal Unadjusted Odds Ratio

Sociologists often want to determine the effect of an exposure on a binary outcome (Kuha and Mills 2020). One example is the effect of parents completing college (*X*) on the offspring completing college (*Y*). A natural way of measuring this effect is to compare the expected college outcomes of children for parents with and without a college degree. An oft-used measure of this effect is the odds ratio,


1a $$OR_{\text{MargUnadj}}=\frac{\frac{\text{Pr}\left(Y=1|X=1\right)}{1-\text{Pr}\left(Y=1|X=1\right)}}{\frac{\text{Pr}\left(Y=1|X=0\right)}{1-\text{Pr}\left(Y=1|X=0\right)}}.$$

The odds ratio measures the effect of *X* on *Y* as the ratio *between* the odds of success relative to failure for one group *and* the odds of success relative to failure for another group. In the college example, the odds ratio gauges how much more likely (stated in odds) children of college-educated parents, compared to children of noncollege-educated parents, are to complete college themselves. The odds ratio in equation (1a) is a population-averaged effect (Zeger et al. 1988). It gauges the population response to a unit change in the exposure. Because the odds ratio is not conditioned on any covariates, we define this effect as a marginal unadjusted odds ratio, where marginal refers to the averaging over the population and where unadjusted refers to the absence of conditioning on other variables.

Often researchers are interested in conditioning the effect of interest on a third variable, *Z*. We term this third variable the *confounder* and assume that it is a binary variable.^1^ Conditioning the odds ratio on a confounder results in three distinct odds ratios whose interpretations differ. In the following paragraphs, we define these three odds ratios and present their respective interpretations.

However, before we do so, for us to compare the marginal unadjusted odds ratio in equation (1a) to the other odds ratios we present below, we rewrite this marginal unadjusted odds ratio as:


1b $$OR_{\text{MargUnadj}}=\frac{\frac{\text{Pr}\left(Y=1|X=1\right)}{1-\text{Pr}\left(Y=1|X=1\right)}}{\frac{\text{Pr}\left(Y=1|X=0\right)}{1-\text{Pr}\left(Y=1|X=0\right)}}=\frac{\frac{\text{Pr}\left(Y=1|X=1,Z=0\right)\text{Pr}\left(Z=0|X=1\right)+\text{Pr}\left(Y=1|X=1,Z=1\right)\text{Pr}\left(Z=1|X=1\right)}{1-\left[\text{Pr}\left(Y=1|X=1,Z=0\right)\text{Pr}\left(Z=0|X=1\right)+\text{Pr}\left(Y=1|X=1,Z=1\right)\text{Pr}\left(Z=1|X=1\right)\right]}}{\frac{\text{Pr}\left(Y=1|X=0,Z=0\right)\text{Pr}\left(Z=0|X=0\right)+\text{Pr}\left(Y=1|X=0,Z=1\right)\text{Pr}\left(Z=1|X=0\right)}{1-\left[\text{Pr}\left(Y=1|X=0,Z=0\right)\text{Pr}\left(Z=0|X=0\right)+\text{Pr}\left(Y=1|X=0,Z=1\right)\text{Pr}\left(Z=1|X=0\right)\right]}}.$$

Because this rewriting is presented in terms of a weighted conditional probability (conditional on the confounder), we can directly compare it to the three odds ratios we present below. This rewriting also enables us to explain how marginal and conditional confounding differ.

### The Conditional Adjusted Odds Ratio

This odds ratio is the adjusted (or controlled) odds ratio that most researchers consider when they condition on a confounder. We present it here in its directly standardized form in which we standardize the odds ratio with respect to the unconditional distribution of the confounder, *Z*:^2^


2 $$OR_{\text{CondAdj}}=\frac{{\left[\frac{\text{Pr}\left(Y=1|X=1,Z=0\right)}{1-\text{Pr}\left(Y=1|X=1,Z=0\right)}\right]}^{\text{Pr}\left(Z=0\right)}}{{\left[\frac{\text{Pr}\left(Y=1|X=0,Z=0\right)}{1-\text{Pr}\left(Y=1|X=0,Z=0\right)}\right]}^{\text{Pr}\left(Z=0\right)}}\frac{{\left[\frac{\text{Pr}\left(Y=1|X=1,Z=1\right)}{1-\text{Pr}\left(Y=1|X=1,Z=1\right)}\right]}^{\text{Pr}\left(Z=1\right)}}{{\left[\frac{\text{Pr}\left(Y=1|X=0,Z=1\right)}{1-\text{Pr}\left(Y=1|X=0,Z=1\right)}\right]}^{\text{Pr}\left(Z=1\right)}}.$$

The odds ratio in equation (2) is a weighted average of the odds ratios in each group defined by *Z*, where the weights are given by *Z*’s distribution.^3^ Thus, the standardization in equation (2) is a standardization of the group-specific odds ratios. This odds ratio is *specific* to the groups of individuals defined by *Z*. Put in slightly different words, we may think about this odds ratio as the effect for a person whose value equals the average value of the confounder, that is, a person who is Pr(*Z* = 1), and as such also depends on the distribution of *Z*. Returning to the college example, now imagine that we want to condition the effect of parents’ college attainment on children’s college attainment on offspring gender and that gender is equally distributed in the population. In this example, the conditional adjusted odds ratio in equation (2) is the average response to the exposure for a person who is 50 percent male and 50 percent female.

### The Marginal Adjusted Odds Ratio

This odds ratio is the adjusted (adjusting for *Z*) odds ratio on average in the population. In contrast to the conditional adjusted odds ratio in equation (2), the standardization on *Z* involved in this odds ratio is a standardization of the conditional *probabilities*, not the odds ratios:


3 $$OR_{\text{MargAdj}}=\frac{\frac{\text{Pr}\left(Y=1|X=1,Z=0\right)\text{Pr}\left(Z=0\right)+\text{Pr}\left(Y=1|X=1,Z=1\right)\text{Pr}\left(Z=1\right)}{1-\left[\text{Pr}\left(Y=1|X=1,Z=0\right)\text{Pr}\left(Z=0\right)+\text{Pr}\left(Y=1|X=1,Z=1\right)\text{Pr}\left(Z=1\right)\right]}}{\frac{\text{Pr}\left(Y=1|X=0,Z=0\right)\text{Pr}\left(Z=0\right)+\text{Pr}\left(Y=1|X=0,Z=1\right)\text{Pr}\left(Z=1\right)}{1-\left[\text{Pr}\left(Y=1|X=0,Z=0\right)\text{Pr}\left(Z=0\right)+\text{Pr}\left(Y=1|X=0,Z=1\right)\text{Pr}\left(Z=1\right)\right]}}.$$

Because equation (3) is a population-averaged effect, we may think about this odds ratio as the adjusted effect for a person picked at random in the population (adjusting for *Z*).^4^ It gauges the adjusted population response to a unit change in the exposure. In the college example, the marginal adjusted odds ratio would be the average response to the exposure in a population made up by 50 percent males and 50 percent females. We may therefore also think about this odds ratio as the average marginal odds ratio (averaging over the population as defined by *Z*).

### Conditional Unadjusted Odds Ratio

This odds ratio is the unadjusted (not adjusting for *Z*) odds ratio that is *specific* to the groups of individuals defined by *Z*:


4 $$OR_{\text{CondUnadj}}=\frac{{\left[\frac{\text{Pr}\left(Y=1|X=1,Z=0\right)}{1-\text{Pr}\left(Y=1|X=1,Z=0\right)}\right]}^{\text{Pr}\left(Z=0|X=1\right)}{\left[\frac{\text{Pr}\left(Y=1|X=1,Z=1\right)}{1-\text{Pr}\left(Y=1|X=1,Z=1\right)}\right]}^{\text{Pr}\left(Z=1|X=1\right)}}{{\left[\frac{\text{Pr}\left(Y=1|X=0,Z=0\right)}{1-\text{Pr}\left(Y=1|X=0,Z=0\right)}\right]}^{\text{Pr}\left(Z=0|X=0\right)}{\left[\frac{\text{Pr}\left(Y=1|X=0,Z=1\right)}{1-\text{Pr}\left(Y=1|X=0,Z=1\right)}\right]}^{\text{Pr}\left(Z=1|X=0\right)}}.$$

In contrast to the conditional adjusted odds ratio in equation (2), this odds ratio is standardized by the distribution of *Z* within levels of *X* (i.e., the conditional distribution of *Z* given *X*). In other words, we “weight back” to the observed distribution of *Z* with respect to *X*. Thus, because we weight to the distribution of the confounder within *X* rather than its average distribution, we are not controlling for confounding. We may consider this the “unconditional” or unadjusted odds ratio for a person who has the average value on the confounder. In the college example, this odds ratio is the unadjusted response to the exposure for a person who is 50 percent male and 50 percent female. The conditional unadjusted odds ratio is conceptually similar to the unadjusted coefficient measured on the scale of the full model in the approach of Karlson et al. (2012) but is more general in that it allows *X* and *Z* to interact.^5^

## Marginal and Conditional Confounding

### Marginal Confounding

Given the definitions of the four odds ratios, we can define two types of confounding: marginal and conditional. Marginal confounding is defined as the ratio between the marginal unadjusted odds ratio in equation (1b) and the marginal adjusted odds ratio in equation (3). Comparing the two equations, we see that marginal confounding is driven by two factors. First, it is driven by the degree of dependence of *X* on *Z*. Whenever *X* and *Z* are statistically independent, we have that


6a $$\text{Pr}\left(Z=0\right)=\text{Pr}\left(Z=0|X=0\right)=\text{Pr}\left(Z=0|X=1\right),$$

6b $$\text{Pr}\left(Z=1\right)=\text{Pr}\left(Z=1|X=0\right)=\text{Pr}\left(Z=1|X=1\right),$$

and, as a consequence, equation (3) collapses to equation (1b). Second, marginal confounding is driven by the degree of dependence of *Y* on *Z* net of *X* (i.e., the conditional effect of *Z* on *Y* net of *X*). If *Z* has no independent or direct effect of *Z* net of *X*, we have that


7a $$\text{Pr}\left(Y=1|X=0,Z=0\right)=\text{Pr}\left(Y=1|X=0,Z=1\right),$$

7b $$\text{Pr}\left(Y=1|X=1,Z=0\right)=\text{Pr}\left(Y=1|X=1,Z=1\right).$$

In other words, the conditional probability of *Y* = 1 given *X* is the same for each level in *Z*. Under this scenario, equation (3) collapses to equation (1b).

How are we to interpret marginal confounding? We suggest that it gauges the extent to which the effect of *X* on *Y* can be explained by *Z on average* in the population. The marginal unadjusted odds ratio is the unit response to *X* on average in the population, whereas the marginal adjusted odds ratio is the directly standardized unit response to *X* on average in the population, adjusting for *Z*. Thus, we may think about marginal confounding in terms of a weighted average of confounding in the population (averaging over the population’s heterogeneity). It is the expected degree of confounding in the population or the degree of confounding we would expect *on average* in a population. In the college example, marginal confounding would be the extent to which gender would confound the association between parents’ and children’s college attainment *on average* in a population made up by 50 percent females and 50 percent males.^6^

### Conditional Confounding

Conditional confounding is defined as the ratio between the conditional unadjusted odds ratio in equation (4) and the conditional adjusted odds ratio in equation (2). Comparing the two equations, we find that conditional confounding is driven by the exact same two factors as marginal confounding. Under the independence assumption in equation (6) and/or the conditional independence assumption in equation (7), equation (2) reduces to equation (4).

Our suggested interpretation of conditional confounding is that it measures the degree of confounding for specific persons defined by the confounder *Z*. The conditional unadjusted odds ratio is the unadjusted odds ratio that is *specific* to the groups of individuals defined by *Z*, whereas the adjusted counterpart is the odds ratio specific to the same groups of individuals. In the college example, conditional confounding gauges the degree to which gender would confound the association between parents’ and children’s college attainment for a person who is 50 percent female and 50 percent male. Thus, we may also think about conditional confounding as confounding for the person who has a mean value on the confounder.

### Quantifying Confounding

Researchers are often either implicitly or explicitly interested in quantifying the degree of confounding or mediation. A common way to report this degree is to express in percent the reduction (or change) in the unadjusted effect after adjusting for a confounder (Karlson et al. 2012).^7^ We follow this convention and report marginal and conditional confounding in terms of percent change. However, we do so using the logarithm to the odds ratio, that is, the *log odds ratio* as this corresponds to the way in which percent confounding or mediation has been reported in the previous literature (e.g., Karlson, Holm, and Breen 2012).

We consequently define the marginal confounding percentage as:


8 $$\frac{\text{ln}\left(OR_{\text{MargUnadj}}\right)-\text{ln}\left(OR_{\text{MargAdj}}\right)}{\text{ln}\left(OR_{\text{MargUnadj}}\right)}\cdot 100,$$

and conditional confounding percentage as:


9 $$\frac{\text{ln}\left(OR_{\text{CondUnadj}}\right)-\text{ln}\left(OR_{\text{CondAdj}}\right)}{\text{ln}\left(OR_{\text{CondUnadj}}\right)}\cdot 100.$$

In the examples we provide in this article, we report this percent explained (or mediated) of the unadjusted (total) effects.

### Simulated Example

Marginal and conditional confounding using logits measure two different types of confounding, that is, population-averaged and subject-specific confounding. Because they measure different quantities and may therefore not yield similar results, we present a stylized example in which the two lead to different results. In the section “Empirical Examples,” we provide two examples using real data.

Table 1 provides the data from a simulation in which marginal and conditional confounding differ. The table shows the cross-tabulation of a binary predictor (*X*) and a binary outcome (*Y*) for the two levels in a binary confounder (*Z*). The example is constructed such that the *X*-*Y* odds ratio is 2.67 for both levels in *Z*, meaning that there is no interaction effect on the logit scale. Still, the effects differ dramatically by the levels in *Z*. For *Z* = 0, the risk ratio, Pr(*Y* = 1|*X* = 1)/Pr(*Y* = 1|*X* = 0), is 2.00, and the differences in conditional probabilities, Pr(*Y* = 1|*X* = 1) − Pr(*Y* = 1|*X* = 0), is 20 percentage points. For *Z* = 1, the corresponding figures are 1.07 and 0.06, mainly because *X* = 1 is a relatively rarer occurrence among those with *Z* = 1 than among those with *Z* = 0.

**Table 1.** Simulated Example.

	*Y* = 0	*Y* = 1	Total
*Z* = 0			
*X* = 0	240	60	300
*X* = 1	150	100	250
Total	390	160	550
*Z* = 1			
*X* = 0	20	180	200
*X* = 1	10	240	250
Total	30	420	450

To measure the degrees of marginal and conditional confounding, we compare equations (1b) and (3) and equations (2) and (4), respectively, by using the percent explained in equations (8) and (9). We find that the marginal confounding percentage is 31.9 and the conditional confounding percentage is 26.8, where we define the confounding percentage as the fraction of the unadjusted odds ratios that can be explained by adjusting for *Z*. The difference is quite substantial: The expected degree of confounding by *Z* on average in the population is close to one-third, whereas the expected degree of confounding by *Z* for a person with a mean value of *Z*, that is, who is 55 percent *Z* = 0 and 45 percent *Z* = 1, is close to one-quarter. Thus, the degree of confounding differs depending on the type—marginal or conditional—of confounding that the researchers will use. Moreover, their interpretation differs in that the marginal confounding percentage has a population-average interpretation, whereas the conditional confounding percentage has a subject-specific interpretation.

## KHB as Conditional Confounding

The method by Karlson et al. (2012) has become a popular method for gauging the extent of confounding in logit and other nonlinear probability models. In this section, we demonstrate that their method recovers a specific type of *conditional* confounding as we define it in this article. The KHB method works under the assumption of no interaction of exposure and confounder (Breen, Karlson, and Holm 2013; Karlson et al. 2012)—an assumption that places certain restrictions on the proportionality of the odds, namely that


10a $$\frac{\text{Pr}\left(Y=1|X=1,Z=0\right)}{1-\text{Pr}\left(Y=1|X=1,Z=0\right)}=k\frac{\text{Pr}\left(Y=1|X=0,Z=0\right)}{1-\text{Pr}\left(Y=1|X=0,Z=0\right)},$$

10b $$\frac{\text{Pr}\left(Y=1|X=1,Z=1\right)}{1-\text{Pr}\left(Y=1|X=1,Z=1\right)}=k\frac{\text{Pr}\left(Y=1|X=0,Z=1\right)}{1-\text{Pr}\left(Y=1|X=0,Z=1\right)},$$

11a $$\frac{\text{Pr}\left(Y=1|X=1,Z=1\right)}{1-\text{Pr}\left(Y=1|X=1,Z=1\right)}=m\frac{\text{Pr}\left(Y=1|X=1,Z=0\right)}{1-\text{Pr}\left(Y=1|X=1,Z=0\right)},$$

11b $$\frac{\text{Pr}\left(Y=1|X=0,Z=1\right)}{1-\text{Pr}\left(Y=1|X=0,Z=1\right)}=m\frac{\text{Pr}\left(Y=1|X=0,Z=0\right)}{1-\text{Pr}\left(Y=1|X=0,Z=0\right)},$$

where *k* and *m* are proportionality factors. Thus, under the no-interaction assumption in equation (10), the conditional adjusted odds ratio in equation (2) reduces to


12 $$OR_{\text{CondAdj}}^{\text{KHB}}=k=\frac{\frac{\text{Pr}\left(Y=1|X=1,Z=0\right)}{1-\text{Pr}\left(Y=1|X=1,Z=0\right)}}{\frac{\text{Pr}\left(Y=1|X=0,Z=0\right)}{1-\text{Pr}\left(Y=1|X=0,Z=0\right)}},$$

and under the assumptions in equations (10) and (11), the conditional unadjusted odds ratio in equation (4) reduces to


13a $$OR_{\text{CondUnadj}}^{\text{KHB}}=km^{\text{Pr}\left(Z=1|X=1\right)-\text{Pr}\left(Z=1|X=0\right)}.$$

Taking the natural logarithm of equation (13a) yields:


13b $$\text{ln}\left(OR_{\text{CondUnadj}}^{\text{KHB}}\right)=\text{ln}\left(k\right)+\theta \text{ln}\left(m\right),$$

where $\theta =\text{Pr}\left(Z=1|X=1\right)-\text{Pr}\left(Z=1|X=0\right)$ is the equivalent of a linear probability regression of *Z* on *X*. The expression in equation (13b) equals the decomposition detailed in Breen et al. (2013), meaning the KHB recovers a specific type of confounding, namely conditional confounding under a no-interaction assumption.

## Inverse Probability Weighting (IPW) and Marginal Confounding

IPW can be used for obtaining an adjusted marginal odds ratio. The weighting works by creating (for a binary exposure, *X*) two counterfactual populations *Y*(1) and *Y*(0) in which observed confounders are balanced across exposure and, for a causal estimate, it is assumed that unobserved confounders are balanced (Hernan and Robins 2020). Formally, the first stage weight is defined as IPW*^X^* = 1/*f*(*X*|*Z*) where *f* is the probability distribution function of *X* given *Z*, and where *X* is the exposure and *Z* the confounder. Usually, this first stage is modeled (for a binary exposure) using a logistic regression of confounders and their interactions on exposure, and the predicted probabilities, *P_i_*, from the model is then used to construct the IPW. The IPW is then given by 1/*P_i_* for *X* = 1 and 1/(1 − *P_i_*) for *X* = 0. The second stage is a simple model of exposure on outcome weighted by the IPW. It is usual in this second stage to estimate robust standard errors.

Table 2 provides a simple example using simulated data. Here, *Z* is unbalanced across *X* (see the column labeled *N*). However, the IPW calculated by the first stage balances *Z* across *X* (see the column IPW × N) at the population mean of *Z* (0.5 in our example) by creating our two counterfactual populations. In these counterfactual populations, we simply calculate *Y*(1) and *Y*(0) from observed outcomes using the second stage. Taking their difference gives an absolute effect of 0.2 while the marginal odds ratio is 2.33. In contrast, the unweighted difference is 0.24 and the unweighted marginal odds ratio is 2.78.

**Table 2.** Simulated Example Showing Inverse Probability Weighting (IPW) Recovering the Adjusted Marginal Odds Ratio.

Exposure	Confounder	*N*	First Stage			Second Stage		Effect
			Pr (*X*|*Z*)	IPW (1/Pr(*X*|*Z*)	IPW × *N*	Pr(*Y* = 1|*X*, *Z*)	Pr(*Y* = 1|*X*) Given Reweighted *N*	Probability Difference: Pr(*Y* = 1|*X* = 1) − Pr(*Y* = 1|*X* = 0)
*X* = 1	*Z* = 1	60	= 60/100 = 0.6	= 1/0.6 = 1.67	= 60 × 1.67 = 100	.8	= 0.8 × 0.5 + 0.6 × 0.5 = 0.7	= 0.7 − 0.5 = 0.2
	*Z* = 0	40	= 40/100 = 0.4	= 1/0.4 = 2.5	= 40 × 2.5 = 100	.6		Adjusted marginal odds ratio
*X* = 0	*Z* = 1	40	= 40/100 = 0.4	= 1/0.4 = 2.5	= 40 × 2.5 = 100	.6	= 0.6 × 0.5 + 0.4 × 0.5 = 0.5	(Pr(*Y* = 1|*X* = 1)/Pr(*Y* = 0|*X* = 1))/(Pr(*Y* = 1|*X* = 0)/Pr(*Y* = 0|*X* = 0))
	*Z* = 0	60	= 60/100 = 0.6	= 1/0.6 = 1.67	= 60 × 1.67 = 100	.4		= (0.7/0.3)/(0.5/0.5) = 2.33

The IPW and the standardization approach are equivalent but implemented differently (Hernan and Robins 2020). The two stages of the IPW are to model the exposure and confounder relationship and then to use the predicted probabilities from this first stage in an unadjusted regression of these probabilities on the outcome in the second stage. Standardization models the outcome exposure relationship adjusting for confounders in the first stage and then weights the results to the population average of the confounders in the second stage. Both methods can incorporate continuous confounders and interactions. Widely used software packages can be used. For example, Stata implements IPW in its *teffects ipw* function while standardization can be accomplished through the appropriate use of the *margins* function following a regression.

## Empirical Examples

### Education and Social Class Mobility

In this example, we analyze the mediating role of education in social class mobility among children born in 1958 in England, Scotland, and Wales using measures of both marginal and conditional confounding. We use data from the National Child Development Study 1958, which follows individuals from birth throughout today. We exploit the rich information on parents’ social class position, when the individuals grew up, and on the individuals’ educational attainment and social class position as adults. In an additional analysis in which we present an example of the IPW-based approach, we also include a measure of the individuals’ cognitive ability at age 11. We measure parents’ and individuals’ social class position with a dummy variable indicating service class membership, we measure individuals’ educational attainment with a dummy indicating higher education attainment, and we measure cognitive ability at age 11 in four quartile groups. Our final sample comprises 10,507 cases. Table 3 presents the descriptive statistics.

**Table 3.** Descriptive Statistics for the National Child Development Study in Percentage.

Fraction of service class origins	24.3
Fraction of service class destinations	40.2
Fraction with a higher education	26.8

*Note:* The cognitive ability variable described in the main text is measured in quantile groups, meaning that each group contains 25 percent of the sample.

Table 4 presents the main results for the first analysis examining the mediating role of education in social mobility. The marginal unadjusted odds ratio is 2.9, meaning that originating in the service class increases the odds of being a member of the service class themselves as adults by a factor of 2.9 compared to not originating in the service class. Using our standardization approach, the adjusted marginal odds ratio is 1.81. Thus controlling for higher education attainment, on average in the population made up by 27 percent higher educated individuals (and 73 percent nonhigher educated individuals), originating in the service class increases the odds of being in the service class as an adult by a factor of 1.8 compared to not originating in the service class. As the final row in Table 4 shows, this is equivalent to a marginal confounding (or mediation) percentage of 44.4, suggesting that education *on average* mediates roughly half of the intergenerational social class association.

**Table 4.** The Mediating Role of Education in Social Class Mobility—Marginal, Conditional, and KHB Odds Ratios and Confounding.

	Interpretation		
	Marginal	Conditional	KHB
Unadjusted	2.91	3.37	3.49
Adjusted	1.81	2.01	1.98
Confounding percentage (log odds ratios)	44.4	42.4	45.4

*Note*: Estimates based on the National Child Development Study. KHB = Karlson, Holm, and Breen.

Table 4 also shows the results for the conditional odds ratios. The magnitude of these odds ratios is, as we would expect, larger than the marginal ones. They represent the unadjusted and adjusted odds ratios for a (hypothetical) person who is 27 percent higher educated (i.e., has the mean value on the confounder). The relationship between the two, as the mediation percentage in the final row of the table shows, is nonetheless very similar to the marginal one: 42.2 percent of the intergenerational social class association is mediated by education for a person who has average educational attainment.

In the final column of Table 4, we also report the KHB odds ratios and mediation percentage. As we would have expected in light of the KHB method recovers conditional, not marginal, odds ratios, the odds ratios are very similar to the conditional ones. They only differ because of the no-interaction assumption of the KHB method. The mediation percentage is slightly larger, 45.4, but does not change the substantial conclusion. In general, all three approaches produce very similar estimates of mediation that is of very similar magnitude, meaning that they lead to the same substantive conclusion about the role of education in social mobility. Nonetheless, in our view, the marginal odds ratios have a more straightforward interpretation given their population-averaged or “marginal” interpretation. Their absolute magnitudes have a meaningful interpretation in this example, suggesting that they may be the better choice if the odds ratios are to be compared between different studies (Kuha and Mills 2020).

A further advantage of using and comparing marginal odds ratios is that the IPW approach readily provides estimates of the adjusted marginal odds ratios in situations in which researchers want to adjust for multiple confounders. To illustrate this, we conducted an additional analysis in which we obtain the adjusted marginal odds ratio controlling for education and cognitive ability using the IPW approach (not reported here). For the logit model generating the inverse probability weight, we make a full interaction specification of the effects of education and ability. Using this approach, we find that the marginal adjusted odds ratio reduces to 1.58, and a corresponding mediation percentage of 57.0. Thus, education and ability mediate a bit more than half of the intergenerational social class association. This additional example also illustrates yet another advantage of using marginal odds ratios: The unadjusted marginal odds ratio does not change as we control for more and more variables, something that is not true for the conditional odds ratios for which the unadjusted conditional odds ratios (as measured by the KHB method) will increase as more and more variance in the outcome is explained. We return to this point in the Discussion section.

### Educational Differences in the Attitude Toward Whites Treating All Groups Equally

In this example, we analyze data from the General Social Survey 2000 (Smith et al. 2019). Respondents are asked whether they think that whites as a group have a commitment to the fair and equal treatment of all groups in society. We use this attitude variable as our dependent variable and recode it from a 7-point Likert-type scale indicating agreement with the statement to a binary outcome indicating *strong disagreement* (1) or *not* (0).^8^ We analyze the effect on this attitude outcome of completing a four-year college degree with a binary black–white race indicator as the confounding variable (affecting both college attainment and the fair treatment attitude). Put differently, we are interested in the extent to which the college gap in whether people find that whites are committed to the fair treatment can be accounted for by a person’s race. In a subsequent analysis, we control for personal income (measured in four overall bins) to see how much of the college gap in the attitude can be explained by both race and income. We omit from the sample respondents whose race is categorized as “other” and who have missing values on at least one variable. We further restrict our sample to those aged 35–59 years in 2000. The final sample comprises 455 respondents.^9^ Table 5 reports the descriptive statistics.

**Table 5.** Descriptive Statistics for the 2000 General Social Survey Sample in Percentage.

Fraction strongly disagreeing with fair treatment attitude	11.4
Fraction completing a four-year college degree	29.5
Fraction blacks	15.4
Respondent’s personal income	
Less than USD 6,999	7.3
USD 7,000–USD 14,999	15.0
USD 15,000–USD 24,999	18.5
More than USD 25,000	59.3

*Note*: *N* = 455. The sample is restricted to blacks and whites aged 35–59.

Table 6 shows the results from the analysis of how much of the college gap in being strongly against the statement that Whites are committed to a fair treatment of all social groups can be explained by race. The odds ratios are all below 1, meaning that college graduates are less likely than noncollege graduates to strongly disagree with the statement. The unadjusted odds ratio equals 0.691, meaning that college decreases the odds of strongly disagreeing with statement by a factor of 0.69. Using our standardization approach, the adjusted marginal odds ratio is 0.879, meaning that on average in the population made up by 30 percent college-educated individuals (and 70 percent noncollege educated individuals), college decreases the odds of strongly agreeing to the statement by a factor of 0.88. This difference between the unadjusted and adjusted odds ratios translates into the marginal confounding percentage of 65.2, suggesting that race *on average* explains roughly two-thirds of the college gap in the fair treatment attitude, a very substantial amount.

**Table 6.** The Confounding Role of Race in the College Gap in Strongly Disagreeing With Whether Whites as a Group Has a Commitment to the Fair and Equal Treatment of All Groups in Society—Marginal, Conditional, and KHB Odds Ratios and Confounding.

	Interpretation		
	Marginal	Conditional	KHB
Unadjusted	0.691	0.655	0.722
Adjusted	0.879	0.770	0.824
Confounding percentage (log odds ratios)	65.2	38.3	40.8

*Note*: *N* = 455. Estimates based on the 2000 round of the General Social Surveys. The sample is restricted to blacks and whites aged 35–59. KHB = Karlson, Holm, and Breen.

Table 6 also shows the results for the corresponding conditional odds ratios. In contrast to the population-averaged interpretation of the marginal counterparts, these quantities represent the unadjusted and adjusted odds ratios for a (hypothetical) person who is 30 percent college-educated. However, the conditional confounding percentage is much lower at around 38 percent, suggesting that race explains less than half of the college gap in the fair treatment attitude for an average person with respect to college attainment. The KHB method reaches a very similar result with a confounding percentage of 41. Thus, in contrast to the education and occupational mobility example, the marginal and conditional confounding percentages in this example are very different and yield different substantive conclusions: On average in the population, race appears to be a powerful confounder, whereas for the average person with respect to college attainment, it is a much less powerful confounder.

To further stress the differences between the two types of confounding, we run a supplementary analysis based on the IPW approach in which we control for both race and the personal income variable (not reported here). Adjusting for both race and income, we obtain a marginal adjusted odds ratio of 0.993, implying that, considered together race and income explain 98 percent (i.e., virtually all) of the college gap in the fair treatment attitude. The corresponding confounding percentage using the KHB approach yields a confounding percentage of merely 66, again leading to very different substantive conclusions. In conclusion, this example shows that confounding (or mediation) percentages obtained via marginal or conditional odds ratios can differ.

## Discussion

This article argues that sociologists should distinguish between marginal and conditional odds ratios when they analyze the confounding or mediating role of control variables for associations of interest. We show that confounding comes in both marginal and conditional forms, we explain how their interpretation differs, and we demonstrate that the KHB method recovers a particular type of conditional confounding under a no-interaction assumption.

While marginal and conditional confounding percentages in most situations arguably would be quite similar, we find the interpretation of the marginal odds ratios to be more intuitive and relevant for a large body of sociological research. Marginal odds ratios are “effects” on average in a population and we suggest to interpreting confounding in the same “population-averaged” way. Indeed, the widely used KHB method recovers conditional, not marginal confounding, and for this reason the absolute magnitude of conditional (subject-specific) odds ratios are difficult to meaningfully interpret (although the relationship between adjusted and unadjusted conditional odds ratios are not). Given that IPW easily provides adjusted marginal odds ratios, even with multiple control variables, sociologist should consider adopting this approach in future work.

Using marginal over conditional odds ratios has the additional advantage that the *unadjusted* marginal odds ratio is fixed and does not change as we adjust for an increasing number of control variables (i.e., it does not depend on the distribution of the confounder). This is not true for the conditional or KHB counterparts for which the unadjusted conditional odds ratios will increase as more and more control variables are adjusted for. This is particularly critical if estimates are to be compared across studies or populations.^10^ In such comparisons, the conditional unadjusted odds ratios could differ as a result of the predictive power of control variables being different in different samples. This is not true of the marginal unadjusted odds ratio. For example, in comparative class mobility research (e.g., Breen and Müller 2020), researchers are primarily interested in (i) comparing odds ratios among countries or cohorts and (ii) comparing the mediating role of educational attainment in these odds ratios. While it is possible to approach these questions from a conditional confounding perspective using the KHB approach (Breen and Karlson 2014), the conditional unadjusted odds ratios will depend directly on the predictive power of education, meaning that comparing the magnitudes of these odds ratios across, say countries, would be highly problematic. Such ambiguity would be resolved if researchers consistently use the marginal “population-average” odds ratio we present in this article.

Nonetheless, one disadvantage of adopting marginal odds ratios is that the elegant decomposition of total effects into their direct and indirect counterparts that apply for conditional odds ratios under the no-interaction assumption (Breen et al. 2013) does not readily apply to the marginal case. Thus, researchers might need to balance the need for detailed decompositions using the conditional odds ratios of the KHB approach and more general decompositions using the marginal odds ratios of a standardization or IPW approach.

While our article suggests a simple standardization approach for the binary logit model, future research should clarify the extent to which our approach extends to the ordered and multinomial logit model. The KHB approach, for example, readily extends to these models, and using IPW to recover marginal confounding should, in principle, work with these types of models. However, the relationship between the IPW approach and our standardization approach in the ordinal and multinomial logit model is not clear and would be a topic worthy of investigation in future research.

## Supplemental Material

Supplemental Material, sj-docx-1-smr-10.1177_0049124121995548 - Marginal and Conditional Confounding Using Logits Click here for additional data file. Supplemental Material, sj-docx-1-smr-10.1177_0049124121995548 for Marginal and Conditional Confounding Using Logits by Kristian Bernt Karlson, Frank Popham and Anders Holm in Sociological Methods & Research

## Notes

1. Depending on the temporal ordering of the variables, *Z* could also be a mediator, mediating the association between the predictor *X* and the outcome *Y*. While the interpretation would differ depending on whether *Z* is a confounder or mediator, the mathematical definitions we use here will not.

2. We later turn to the conditional odds ratio that is obtained from a logit model not involving an interaction term between the predictor and confounder. See the section “KHB as Conditional Confounding.”

3. In Online Appendix A1 (which can be found at http://smr.sagepub.com/supplemental/), we show how equation (2) can be interpreted in terms of the more familiar log odds ratios (logit regression coefficients).

4. In Online Appendix A2 (which can be found at http://smr.sagepub.com/supplemental/), we show how this odds ratio can be interpreted in terms of coefficients from a linear probability model.

5. We show in Online Appendix A3 (which can be found at http://smr.sagepub.com/supplemental/) how this conditional unadjusted log odds ratio can be expressed in terms of logit regression coefficients and log odds ratios that, respectively, capture the correlation between *X* and *Z* and the effect of *Z* on *Y*.

6. We realize that gender cannot be a true confounder of the association between parents’ and children’s college attainment, as parental college attainment and child gender are independent of each other in most countries. Nonetheless, we believe that this example provides a good intuition about how to think about the different types of confounding.

7. Formally, this percentage is calculated as the *difference* between the unadjusted and adjusted effects *divided by* the unadjusted effect.

8. We recode values 6 and 7 into a strong disagreement category (1) and values 1–5 into an agreement category (0). We realize that slicing the ordinal response in this way is arbitrary and that results most likely would have been different, had we chosen another cutoff. However, given its expositional power, we choose only to report results using this cutoff. Moreover, because we could have analyzed the outcome in its ordinal form, in the Discussion section, we briefly discuss how future research should consider extending our approach to the ordered logit model.

9. We realize that the sample is relatively small. However, because we find that the conclusions from this example very clearly show the difference between the two types of confounding, we include it here for expositional and pedagogical reasons.

10. This is also critical in replications of studies in which a replication study might use a slightly different set of covariates.
