# Supplementary material for: Marginal and Conditional Confounding Using Logits
Source: Sociol Methods Res. 2021 Apr 9;52(4):1765–84. doi: 10.1177/0049124121995548 (PMC7615235; doi:10.1177/0049124121995548)
Supplement: Supplemental Material, sj-docx-1-smr-10.1177_0049124121995548 - Marginal and Conditional Confounding Using Logits [file sj-docx-1-smr-10.1177_0049124121995548.docx]

# Appendix A1

For ease of interpretation, we rewrite Eq. (2) in more familiar log odds ratio terms as

where $\beta_{X|Z=z}$is the conditional log odds ratio effect of X on Y (when Z = z) and $\beta_{XZ=1}$ is the interaction term on the log odds ratio scale defined as the difference between the log odds ratios of X on Y for Z = 1 and Z = 0.

# Appendix A2

We rewrite the marginal adjusted odds ratio in Eq. (2) as

where *a* is the probability of Y = 1 when both X and Z equal zero (the intercept in a fully saturated linear probability model), $b_{X|Z=0}$ is the linear probability regression coefficient of Y on X when Z = 0 and vice versa for $b_{Z|X=0}$, and $b_{XZ}$is the interaction effect between X and Z on Y in the linear probability model. From this re-expression, we see that the marginal adjusted odds ratio is a function of the linear probability regression coefficient of X on Y but adjusted for effect of Z on Y and the interaction effect between X and Z on Y.

# Appendix A3

From Eq. (4) we have that

where $\theta$ is the linear probability regression coefficent from regressing Z on X. Taking logs and rearraing yields:

When X and Z are not associated $(\text{i.e., }\theta=0)$ or Z has no effect on Y $(\text{i.e., }\beta_{Z|X=0}=0 \text{ )}$, the conditional unadjusted is equal to the conditional adjusted. Thus, this comparison is not subject to rescaling of logit coefficients across models with and without Z.
